# Right Lateral Femoral Cutaneous Nerve Neuropathy After Surgery for Perforated Appendicitis: A Case Report

**DOI:** 10.7759/cureus.109551

**Published:** 2026-05-24

**Authors:** Patrick Dahdouh, Sarah M Gilyard, Yih-Dar Nien, Michael W Robinson

**Affiliations:** 1 Department of Kinesiology, California State University, Northridge, Los Angeles, USA; 2 Department of Surgery, David Geffen School of Medicine at UCLA (University of California Los Angeles), Los Angeles, USA

**Keywords:** lateral femoral cutaneous nerve, meralgia paresthetica, obesity and diabetes, peripheral sensory neuropathy, ruptured appendicitis, sensory neuropathy, type-2 diabetes mellitus

## Abstract

Perforated appendicitis can be associated with a variety of postoperative complications, most notably abscess and wound infection. Perforation of the appendix can cause regional inflammation that affects neighboring tissues and organs. Occasionally, unexpected additional pathology emerges in the postoperative setting. Due to the retroperitoneal nature of the lateral femoral cutaneous nerve of the thigh (LFCN), resulting inflammation can contribute to neuropathy, known as meralgia paresthetica (MP). The findings of MP are suggested by physical examination. This report describes the case of a patient who presented with perforated appendicitis and abscess formation. Symptoms of right anterolateral pain and numbness developed soon after completion of a laparoscopic appendectomy. The patient had a relatively uncomplicated postoperative course but continued to have neuropathic symptoms two weeks after surgery. These symptoms ultimately resolved spontaneously after approximately six weeks.

## Introduction

Meralgia paresthetica (MP) is a neuropathic condition characterized by numbness, tingling, and burning sensations in the anterior and lateral thigh [1]. It can be caused by inflammation and impingement of the lateral femoral cutaneous nerve (LFCN) and manifest with symptoms such as paresthesia and dysesthesia [2]. Even though electromyography (EMG) and nerve conduction studies may assist with its diagnosis, MP is a complex condition prevalent across several age groups, with multiple etiologies, and is more frequently diagnosed via physical examination. 

The origin of the LFCN explains the potential for involvement in an appendectomy. The nerve originates from the lumbar plexus, specifically the L2 and L3 nerve roots [1]. It emerges as a sensory branch along the lateral aspect of the psoas major muscle before crossing the medial side of the iliacus muscle toward the anterior superior iliac spine (ASIS) [3]. The nerve then passes deep to the inguinal ligament before branching further [3]. Due to the complex nature of MP, it is often misdiagnosed, as its symptoms can overlap with conditions such as pelvic tumors, neuropathies, and others [2]. Risk factors for MP include diabetes mellitus, obesity, pregnancy, and tight-fitting clothing [2]. Inflammation and swelling near the psoas muscle can potentially explain the involvement of the LFCN in an operative setting. 

Although MP is not a well-documented postoperative complication associated with perforated appendicitis, it is important to recognize the potential for nerve injury, particularly in patients with risk factors that may predispose them to nerve involvement. While rare, acknowledging and diagnosing this condition in the early postoperative period can help prevent potentially catastrophic complications such as traumatic fall or surgical drain dislodgement if implemented. This report presents a unique case and discusses both appendicitis and MP in a patient with multiple risk factors.

## Case presentation

A 70-year-old male patient presented with five days of abdominal pain and nausea. His review of systems was negative for any neuropathic symptoms, specifically lower extremity neuropathy. His past medical history consisted of insulin-dependent diabetes mellitus, obesity with a body mass index (BMI) of 31 kg/m^2^, myocardial infarction, and hypertension. His physical examination was notable for severe right lower quadrant abdominal pain with evidence of focal right lower quadrant peritonitis. He was alert and oriented, and his neurological examination was within normal limits. His labs were notable for a white blood cell (WBC) count of 12,300 k/uL (reference, 4.23-9.07 k/uL). The glucose level was significantly elevated at 265 mg/dL (reference, 110-140 mg/dL) on presentation, and glycated hemoglobin (HbA1C) was 7.7% (reference, < 5.7%). 

A computed tomography (CT) scan was done, demonstrating a 16 mm appendix with intraluminal fecalith and inflammatory fat stranding (Figure 1). During the operation, the patient was noted to have perforated appendicitis with small bowel fibrin deposits and feculent peritonitis (Figure 2). The perforation was contained within the right lower quadrant, ostensibly adjacent to the psoas muscle (Figure 3). The appendix was resected, the purulent fluid was irrigated and washed out from the abdomen, and a drain was left at the appendiceal stump. The patient tolerated the operation well, without the development of severe sepsis or septic shock. Pathology demonstrated acute appendicitis and peri-appendicitis with frank perforation at the base of the appendix.

**Figure 1:**
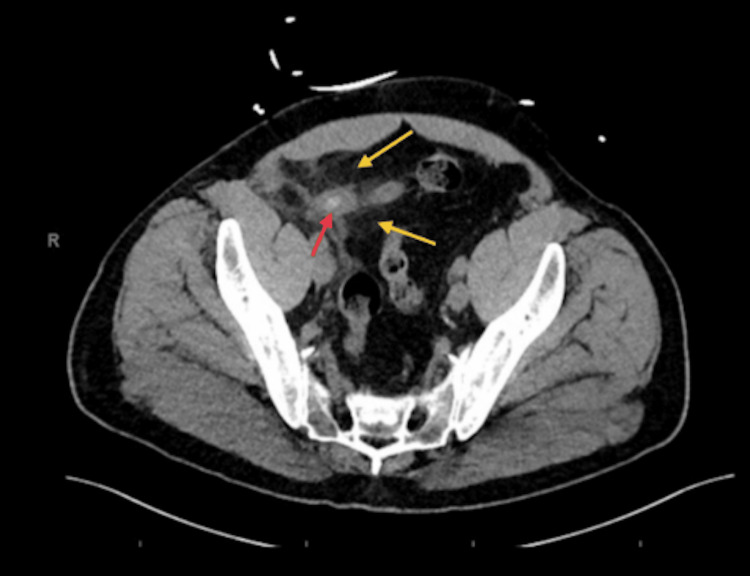
Preoperative CT (transverse cross-section) displaying an affected 16 mm appendix (red arrow) alongside inflamed fat stranding (yellow arrows)

**Figure 2:**
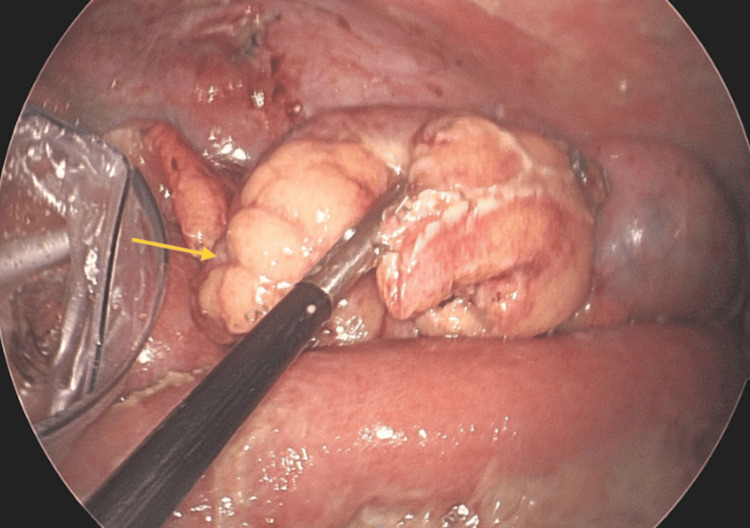
Intraoperative image displaying resection of appendix (yellow arrow) from cecum

**Figure 3:**
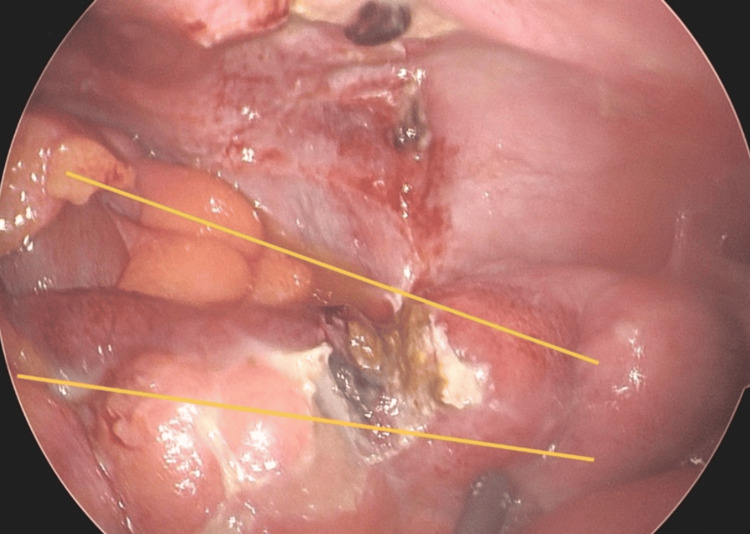
Intraoperative image during appendectomy with approximate location of right psoas major muscle

The patient had a hospital stay complicated by acute kidney injury (AKI) and postoperative ileus, both of which resolved on postoperative day (POD) 3. He complained of distention in the absence of abdominal pain and described right anterolateral thigh numbness from his hip to his mid-thigh. There was no dysesthesia, motor weakness, or neuropathic pain. The neuropathic symptoms started on POD 1. The patient described the numbness as a 7/10 in discomfort, once again with no complaints of pain in the right anterolateral thigh. His ileus resolved spontaneously without procedural intervention. A focused neurological examination revealed intact motor function without deficits and no additional sensory neurolpathical abnormalities. On POD 5, he still noted the neuropathic symptoms but did quite well from an infectious and inflammatory standpoint and was stable for discharge. 

On a follow-up on POD 15, the patient was quite well from an abdominal perspective. However, he still complained of numbness in the right anterior thigh, albeit somewhat lessened in intensity. He described the discomfort as 3/10 in intensity. There was no pain or dysesthesia and his neuropathy did not affect his ambulation or activity level. A neurology outpatient consultation was placed, but he was told that his symptoms would likely improve over time without intervention. On a follow-up on POD 41, the patient reported that all neuropathy symptoms had resolved. 

## Discussion

MP, also known as Bernhardt-Roth Syndrome, is a rare condition [2]. It is characterized by distinctive paresthesia to the anterior and lateral aspects of the thigh (Figure 4) [3]. The pathophysiology of MP is the result of compression of the LFCN [2-4]. Data suggested its occurrence in 10.7 per 100,000 men and 13.2 per 100,000 women [4].

**Figure 4:**
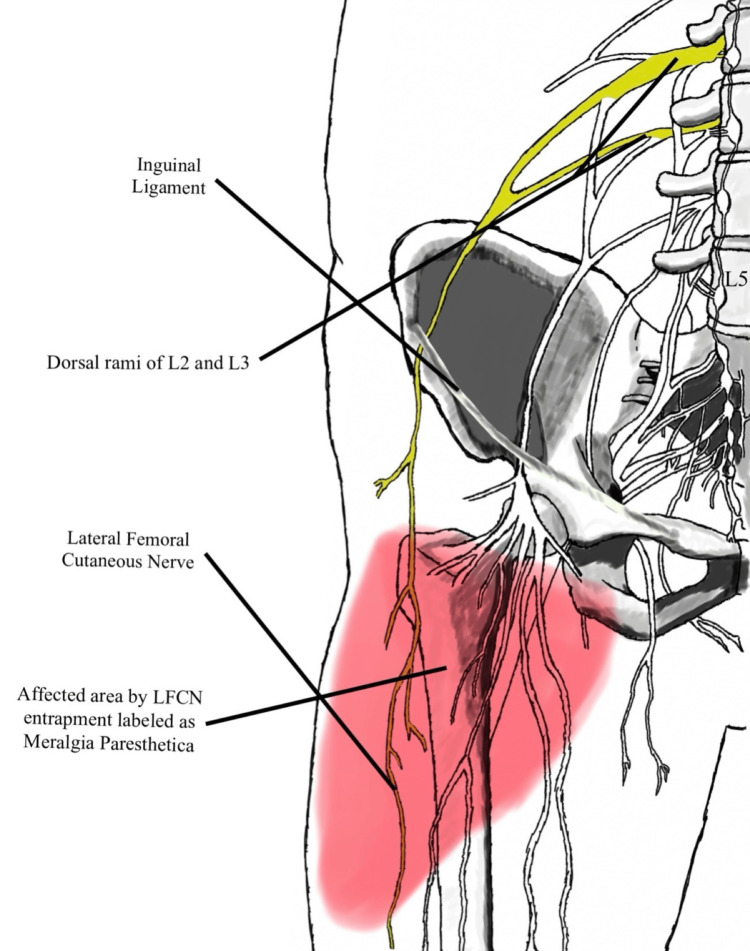
Illustration of the LFCN anatomy and the patient's affected region of neuropathy LFCN: lateral femoral cutaneous nerve Image Credit: Patrick Dahdouh (author); created with Freeform application (Apple Inc., Cupertino, California, United States)

Physical examination is the primary diagnostic method. Advanced diagnostic modalities include EMG, nerve conduction studies, and ultrasound-guided direct corticosteroid nerve injections. There can be a high variability of the LFCN in relation to its smaller branch divisions, making diagnosis with ultrasound challenging. However, some studies report a sensitivity and specificity of 87-95% and 90-95%, respectively [5]. Electrophysiological tests can be particularly technically challenging and therefore not always conclusive, secondary to factors such as obesity. Management of MP is often guided by a neurologist, although symptoms are frequently self-limited. Therefore, it is vital to treat the underlying risk factors of a patient. Treatment of these factors may lower the incidence rate of MP. Non-surgical treatment includes a trial of lifestyle modifications, such as weight loss and avoiding compressive clothing, as well as medications including non-steroidal anti-inflammatory drugs (NSAIDs), muscle relaxants, and tricyclic antidepressants (TCAs). Additional options include ultrasound-guided nerve injections with anesthetics and/or corticosteroids, as well as transcutaneous electrical nerve stimulation (TENS). Surgical treatment remains controversial but may involve decompression and/or neurectomy of the LFCN [4]. 

Multiple etiologies can contribute to MP, which can obscure a single definitive cause for providers. In the present case, several factors may explain the potential development of MP. These include the patient’s obesity and prior history of diabetes mellitus, as well as potential inflammatory impingement of the nerve secondary to the perforated appendix. Obesity increases pressure across the inguinal ligament, where the LFCN courses deep, and may result in nerve impingement (Figure 4). In contrast, diabetes mellitus may affect the LFCN through metabolic mechanisms. Disruption of the sorbitol pathway in diabetic patients can contribute to peripheral neuropathy, including LFCN neuropathy [5].

BMI appears to be a positive indicator for MP. In a 10-year population survey, the average incidence initially occurred at a mean age of 50 years with an average BMI of 28.7 kg/m² (WHO “pre-obesity”). During the five-year study period, both the mean age and BMI increased to 53 years and 30.9 kg/m² (WHO obesity category), respectively [6,7]. Diabetes mellitus is also a relevant risk factor. Mean BMI is higher in individuals with diabetes mellitus, and this leads to the surmise that there is an association between diabetes mellitus and MP. Notably, in the study by Parisi et al., the incidence of MP was 247 per 100,000 patient years, which is seven times higher than the incidence in the general population [6], thus confirming the surmise.

In this case, the patient had a significant infectious and inflammatory condition with underlying risk factors of obesity and diabetes, as well as advanced age. He also had an absence of upper leg nerve neuropathy or paresthesia prior to surgery. There were no obvious iatrogenic injuries during his surgery to explain the onset of this patient’s MP. Thus, the development of MP is attributed to retroperitoneal swelling near the site of perforation; however, causality cannot be definitely determined due to the risk factors present. This case thus report highlights the potential development of MP in patients with relevant risk factors, especially since most existing data on post-surgical MP pertains to patients who have undergone orthopedic procedures [5]. Due to the postoperative resolution of the patient's neuropathical symptoms, further neurological tests and evaluation were not ordered. If these symptoms did not abate, a neurological consultation and nerve conduction tests would have been indicated. 

## Conclusions

Perforated appendicitis is associated with a significant inflammatory response, both systemically and within the abdominal cavity. In rare cases, unforeseen postoperative complications may arise. MP, a neuropathicall condition, may present with symptoms including dysesthesia, paresthesia, and other neuropathical manifestations. These symptoms can be evaluated for diagnosis through means such as physical examination, ultrasound, and EMG studies.

Due to a lack of randomized controlled trials on the subject, establishing definite causality between the patient’s risk factors, the surgical procedure, and the subsequent development of MP is difficult. However, the patient’s symptoms strongly suggest the development of this condition. Therefore, MP should be considered in patients with significant intra-abdominal or retroperitoneal inflammation, particularly in the presence of associated risk factors such as obesity and diabetes mellitus. Management of these risk factors may help reduce the incidence of MP and improve quality of life. Treatment of MP is primarily supportive and rarely requires invasive procedures or interventions. Surgical management remains an option for refractory cases, although its role remains controversial.
